# ClipAssistNet: bringing real-time safety feedback to operating rooms

**DOI:** 10.1007/s11548-021-02441-x

**Published:** 2021-07-23

**Authors:** Florian Aspart, Jon L. Bolmgren, Joël L. Lavanchy, Guido Beldi, Michael S. Woods, Nicolas Padoy, Enes Hosgor

**Affiliations:** 1 Caresyntax GmbH, Komturstraße 18A, 12099 Berlin, Germany; 2 Department of Visceral Surgery and Medicine, Inselspital, Bern University Hospital, University of Bern, 3010 Bern, Switzerland; 3 ICube, University of Strasbourg, CNRS, IHU, Strasbourg, France

**Keywords:** Surgical intelligence, Intraoperative safety feedback, Surgical instrument visibility, Laparoscopic Cholecystectomy, Deep learning

## Abstract

**Purpose:**

Cholecystectomy is one of the most common laparoscopic procedures. A critical phase of laparoscopic cholecystectomy consists in clipping the cystic duct and artery before cutting them. Surgeons can improve the clipping safety by ensuring full visibility of the clipper, while enclosing the artery or the duct with the clip applier jaws. This can prevent unintentional interaction with neighboring tissues or clip misplacement. In this article, we present a novel real-time feedback to ensure safe visibility of the instrument during this critical phase. This feedback incites surgeons to keep the tip of their clip applier visible while operating.

**Methods:**

We present a new dataset of 300 laparoscopic cholecystectomy videos with frame-wise annotation of clipper tip visibility. We further present ClipAssistNet, a neural network-based image classifier which detects the clipper tip visibility in single frames. ClipAssistNet ensembles predictions from 5 neural networks trained on different subsets of the dataset.

**Results:**

Our model learns to classify the clipper tip visibility by detecting its presence in the image. Measured on a separate test set, ClipAssistNet classifies the clipper tip visibility with an AUROC of 0.9107, and 66.15% specificity at 95% sensitivity. Additionally, it can perform real-time inference (16 FPS) on an embedded computing board; this enables its deployment in operating room settings.

**Conclusion:**

This work presents a new application of computer-assisted surgery for laparoscopic cholecystectomy, namely real-time feedback on adequate visibility of the clip applier. We believe this feedback can increase surgeons’ attentiveness when departing from safe visibility during the critical clipping of the cystic duct and artery.

**Supplementary Information:**

The online version supplementary material available at 10.1007/s11548-021-02441-x.

## Introduction

Laparoscopic surgeries are often preferred to open interventions. They show a lower incidence of complications and quicker patient recuperation [39]. Nevertheless, laparoscopic interventions come with the challenge of handling the camera, in addition to the skills required for manipulating the instruments. Globally, surgical skills have been clearly associated with patient outcomes [2, 33]. In fact, inadequate visualization is a recognized cause of technical complications and near miss events, e.g., bleeding [5]. Poor visibility of the instrument with respect to the surrounding tissues can result in unintended injury, often not recognized at the time of the procedure. Proper handling of the laparoscopic camera can be used to measure surgeon skills [34].

Among laparoscopic interventions, cholecystectomy is one of the most common. A critical phase of laparoscopic cholecystectomy consists in clipping the cystic duct and artery before cutting (see [32] for a brief description of laparoscopic cholecystectomy procedures). Proper cutting of these structures requires dissection, as well as visual confirmation that the cystic duct/artery are completely captured within the clip applier jaws (see examples in Video SI. 2 in Online resources). Indeed, ensuring full visibility of the distal-most tips of the clip applier enables (i) controlling the structure being clipped (to prevent hemorrhage or a bile leak due to misplaced clips [12, 37, 38]), or (ii) avoiding “past-pointing”: unintentional clipping of neighboring tissues (e.g., the common bile duct [26]).

Despite the risks associated with the lack of visibility during clipping, we still observe surgeons operating with poor visibility (see Video SI. 1 in Online resources for some examples). The clipping phase can in fact be considered as a proxy for rating surgical skills [20]. The existence of complications related to the procedure itself (0.5-3% of patients present cystic duct leakage following LC, up to 7% for patients with complicated gallstone disease [11]) shows the need for new approaches to surgical safety.

In this work, we present a novel intra-operative safety feedback mechanism during the critical clipping phase. Similar to lane departure warning systems during car driving (see [40] analogy), we propose to warn surgeons when departing from safe behavior, i.e., from good visibility. Specifically, this feedback alerts surgeons when the tip of the clip applier is not adequately visualized, indicating a potentially unsafe situation.

We approach this problem by building a binary image classifier which detects the clipper tip visibility. To this end, we utilize a new dataset tailormade for our application. This dataset is composed of laparoscopy cholecystectomy videos with frame-wise binary labels for the clipper tip visibility. We further present ClipAssistNet. This image classifier ensembles predictions of 5 residual neural network classifiers [15] trained on different subsets of our dataset.

Given the inherent availability of endoscopic recordings, laparoscopy has been an ideal candidate for computer vision research. At the video level, particular efforts were put on video segmentation for surgical phase [4, 9, 18, 35] and surgical action recognition [13, 21], as well as in remaining surgery duration prediction [27, 36]. Importantly, notable advances were also achieved at the frame-level on instrument detection [17, 18, 22] (see [6] for a review of earlier works), as well as in pixel-wise semantic segmentation of surgical images [3, 14, 16, 23, 24, 29].

Unlike our present application, previous studies on instrument detection and image segmentation did not address the full visibility of the instrument present in the frame. Instead, they focused on detecting pixels corresponding to a given instrument, ignoring its non-visible (e.g., occluded) parts. Similarly, studies on instrument pose estimation [1, 8, 19] are usually extracting the visible keypoints of the instrument, ignoring its non-visible physical end. As these approaches are supervised and require tedious manual annotations, we approach tip visibility detection as an image classification problem, which does not require any spatial annotation. Analysis of the saliency maps shows that the ClipAssistNet focuses on the correct parts of the image, that is, the tip of the clip applier.

In summary, we contribute to the field of computer-assisted surgery by proposing a novel computer vision application for safe laparoscopy cholecystectomy. To this end, we introduce a corresponding dataset and the accompanying model to detect clipper tip visibility.

## Material and methods

### Data

Our model relies on frame-wise binary labels of the clipper tip visibility. A frame is labeled as tip visible if the tip of the clip applier is present in the frame and if it is not occluded by another tool or tissue.

Clippers are composed of a shaft and two jaws; we define the clipper tip as the tips of both jaws. In an open position, the instrument tip is labeled as visible if and only if the tips of both jaws are not occluded.

**Fig. 1:**
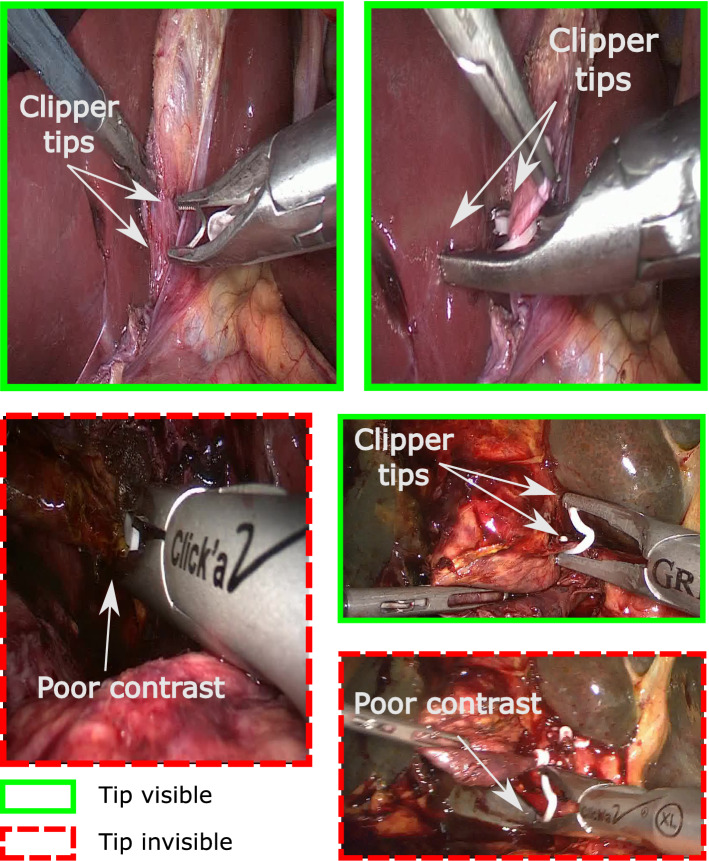
**Individual video frames are annotated for clipper tip visibility.** The framing color codes the label of each image, that is, (green solid line) clipper tip visible and (red dashed line) clipper tip invisible. The top images display the two different types of clip appliers: (left) metal clip and (right) polymer clip

Our data include appliers for metal clips (mostly from the brand Aesculap Challenger®) and for polymer clips (from the brand Grena®) (respectively, top left and right in Fig. 1). In case of the polymer clip applier, we consider the visibility of the polymer clip tip as sufficient (see Fig. 1, right center image).

The frames are annotated in their appearance order, that is, consecutive frames are annotated successively. Nevertheless, the annotators are asked to label each frames independently of the context, i.e., of the surrounding frames. In other words, we consider as non-visible the cases in which we are not able to conclude while looking at the sole frame. For example, the following cases are annotated as non-visible:

1. only the tip of the clipper is present in the frames, i.e., one cannot recognize a clipper in this frame;
2. the clipper is subject to strong motion blur, i.e., one cannot identify the clipper or its tip;
3. the contrast is poor, i.e., one cannot determine if the tip is occluded or not based on this single frame.

This context-independent annotation strategy is designed to reflect the final use case: surgeons should not clip in case of poor visibility (e.g., bad contrast).

Using this annotation strategy, we build a new dataset containing 300 non-robotic laparoscopic cholecystectomy video recordings from the Inselspital (the University Hospital of Bern). We sample these videos at 5FPS and annotate all frames containing at least a few pixels of the clipper. This includes frames in which one cannot recognize the clipper given this sole frame.

In the following, we split the data into a “*training/validation*” set and a test set (frames from 29 videos). We use the *training/validation* set for model training and selection using a cross-validation scheme. The test set is used to evaluate the performance of the final model selected using cross-validation on the *training/validation* set.

To compare annotations across annotators and also obtain a consistent test set for evaluating ClipAssistNet reliably on the end application, the 29 test videos are annotated by three different annotators (see Table 1 for a summary). The remaining videos, used in the *training/validation* set, are annotated by single annotators.

**Table 1:** Summary of the annotations

Dataset	Video	Annotation	Total Frame	Tip visibility	
	count	count per frame	count	Visible	Invisible
*Training/validation*	271	1	111154	35.6%	64.4%
Test	29	3	11316	37.4%	62.6%

For the cross-validation, we partition the *training/validation* set by selecting videos using a deterministic algorithm. Indeed, due to differences in the video length, using a random sampling approach for the partition would lead to highly unbalanced partitions size in terms of frame count. Instead, after sorting them by decreasing frame counts, videos are assigned one by one to the subset with the lowest frame count.

### Modeling

We follow a data-driven approach to the tip-visibility classification. In particular, we train a deep convolutional neural network model to classify the visibility of the clipper tip in each frame.

#### Models

We opt for a Resnet50-based [15] neural network architecture with weights pre-trained on ImageNet. Images are resized to 224x224 pixels and normalized using the ImageNet statistics. During training, data augmentation is applied on the training images. The validation set is monitored for early stopping and model checkpointing (see Sect. 2.2.2 for implementation details).

The tip visible/invisible labels are slightly imbalanced in our dataset (Table 1). Additionally, our training samples (images) are not identically independently distributed. Samples coming from a single video might be more similar than images coming from different videos. Combined with highly variable annotated frame counts per videos (ranging from 62 to 1693 frames per videos, meanstd: 271252), this may potentially bias our algorithm toward videos with high number of frames.

To address these peculiarities, we compare different variants of the binary cross-entropy (BCE) loss. Besides standard BCE and class-weighted BCE, we also consider a video-weighted cross-entropy loss which accounts for different frame counts per video. This video-weighted loss increases the emphasis on videos with a low number of frames, i.e., with a short clipping phase or a lower sampling rate. The loss of a training batch of size, *p*, is defined as:

1 $$\begin{aligned} \mathrm{loss}= & {} - \sum _{i=0}^{p} w_{\mathrm {video}(i)} (y_i \log ({\hat{y}}_i)\nonumber \\&+ (1-y_i) \log (1-{\hat{y}}_i)) \end{aligned}$$

where $w_{\mathrm {video}(i)}$ is the weight of the video sample *i* is originating from. ${\hat{y}}_i$, $y_i$ are, respectively, the model confidence output and the ground truth label for sample *i*. We set the weights of each video *j*, $w_{j}$, as the inverse of the training sample count from this video: $w_{j} = \frac{1}{|\mathrm {video}_j|}$.

Moreover, our training data contain a certain amount of labeling noise due to the difficulty of annotating frames with poor contrast (see Sect. 3.1 for details). For comparison sake, we evaluate whether this noise can be accounted for by implementing a forward label noise correction scheme [25]. This scheme consists in multiplying the network predictions by the label confusion matrix before computing the loss.

To improve the model accuracy, we ensemble the five network models trained during the cross-validation on the *training/validation* set. The models share the same architecture and loss but are trained on the different folds of the *training/validation* dataset. The models are ensembled using weighted average of the predicted confidence. We name the resulting model *ClipAssistNet*. We evaluate the ClipAssistNet performance on the test set.

#### Implementation details

The models are trained using Pytorch Lightning and pretrained weights of the Resnet50 convolutions are loaded from torchvision (v0.6.0). The classification head consists of an adaptive 2D average pooling layer followed by a dense layer (input size 2048, output size 2).

Batches of 64 frames are used with gradient accumulation over 5 batches. The networks are trained using a plateau learning rate scheduler reducing on the validation loss (Pytorch’s ReduceLROnPlateau scheduler). The scheduler has a patience of 5 epochs and a reduction factor of 0.8. The initial learning rate is chosen for each loss using the learning rate finder algorithm implemented in Pytorch Lightning. This algorithm selects the optimal learning rate by considering solely the training data [31]. The used initial learning rates are 1.98e-4 for the BCE loss, 1.51e-4 for the class-weighted BCE loss, 1.21e-4 for the video-weighted BCE loss and 1.01e-4 for the BCE loss with label-noise correction.

Early stopping is used with a patience of 10 epochs monitoring the Area Under the Receiving Operator Curve (AUROC) on the validation set. The maximum of 80 epochs is never reached. For testing, we use the model checkpoints corresponding to the highest AUROC on the validation set.

Image preprocessing is performed using the albumentations library [7]. Data augmentation includes random rotation (40), scaling (30%) and translation (20%), as well as brightness (20%) and contrast (from -20% to 25%) change. Each transformation has a probability of 0.75. Where needed, we use reflection to fill the missing values at the border.

## Results

### Annotation consistency analysis

A significant contribution of the present work lies in the definition of the application and the corresponding annotation guidelines (see description in Sect. 2). Despite our efforts to define an objective guideline, the frame-wise tip visibility labels stay subjective in edge cases. Particularly in case of poor contrast frames, defining the tip visibility remains at the discretion of the annotator. This results in divergence of labels for given frames across different annotators.

To reduce the labeling error in our test set, we triple annotated our whole test set and use the prevailing label for each image. Overall, annotators do not meet a full agreement on 18% of the triply annotated frames. That is, for these frames at least one annotator disagrees with the 2 others on whether the tip is visible or not. These frames correspond to edge cases in which the image contrast is low or the tip of the clip applier is close to the tissue. This high disagreement rate reflects the difficulty to label certain frames.

In addition, annotators have an average error rate of 6.8%. Annotator error rate is defined as the percentage of the frames a given annotator disagrees with the two other annotators. In other words, if the test set is single annotated (as in the training dataset), 6.8% of the frame labels might be incorrect.

### Modeling

We keep safety in focus when deciding which model performance metrics to track. Specifically, we want to ensure that the model catches as many tip invisible cases as possible. We therefore define the tip invisible cases as our positive cases, tip visible labels/predictions being the negative cases. This is the case for all reported metrics.

Our binary classifier predicts a confidence on the instrument tip being visible or not. Binary predictions are obtained by setting a threshold on this confidence. We consider both threshold-independent and threshold-dependent performance metrics, namely the area under the curve of the receiving operator (AUROC) and the specificity at 95% sensitivity. The latter metric corresponds to the model specificity (i.e., true negative rates) when setting a confidence threshold which corresponds to a sensitivity (i.e., true positive rate) of 95%.

To begin with, we train a Resnet50-based neural network with a simple binary cross-entropy (BCE) loss on a single split (80/20) of the *training/validation* dataset. The model classifies the tip visibility on the validation set with an AUROC of 0.89 and a specificity (at 95% sensitivity) of 61%.

**Fig. 2:**
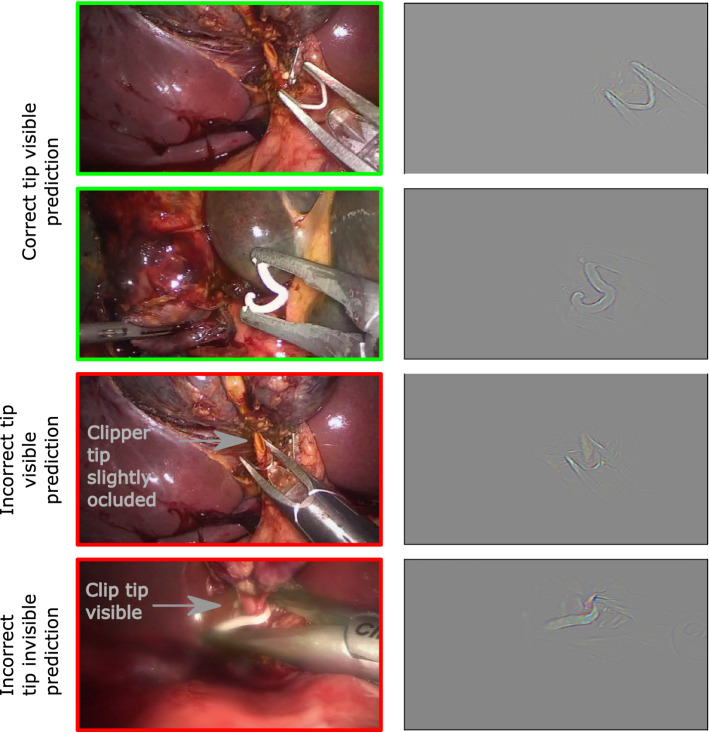
**The model learns to recognize the shape of the clipper but fails when the tip is partly occluded or visible.** Original images (left) and the corresponding class activation maps, i.e., guided gradCam (right), obtained with a single Resnet50 classifier with BCE loss. The colored frame around the original images encodes whether the prediction is (green) correct or (red) incorrect

Looking at the saliency maps (Guided grad-CAM [30]) in Fig. 2, we can see that the model learns to recognize the clipper shape. Nevertheless, the model has difficulty in edge cases, such as when the tip is slightly occluded (Fig. 2, third row) or slightly visible (Fig. 2, 4th row).

Additionally, 45 out of the 50 highest loss-frames correspond to tip visible labels, while 42 out of the 50 lowest loss-frames are tip invisible labels. This could be due to the class imbalance toward tip invisibility in the training dataset.

We further compare the impact of different losses on the classification performance of the Resnet50-based classifier. Each loss is chosen to adapt our model to a given peculiarity of our dataset. Specifically, we try to account for:

1. class imbalance, by using class weights inversely proportional to the class presence in the training set;
2. label noise, by using the forward labeling noise correction methods presented by Patrini et al. [25];
3. disparity in the frame counts per videos, by setting samples’ weights inversely proportional to the frame counts from the video they are issued from (see Sect. 2 for details).

Implementation details are described in Sect. 2.2. The performance improvements are measured using a fivefold cross-validation on the *training/validation* set.

For all tracked metrics, the model performance is the highest when using the simple binary cross-entropy loss (Table 2). Yet, the difference is not significant.

**Table 2:** **Performance of the models using different losses measured through cross-validation.**The table reports the mean and standard deviation of the metrics measured on the validation sets during cross-validation on the *training/validation* dataset

	AUROC	Specificity at 95%sensitivity
BCE loss	0.8916±0.0075	0.5813±0.0187
BCE loss with class weights	0.8911±0.0081	0.5746±0.0250
BCE loss with label noise correction	0.8857±0.0088	0.5741±0.0312
BCE loss with video weights (frame counts per videos)	0.8860±0.0096	0.5682±0.0295

To best use the available data, we ensemble the 5 models trained with the standard binary cross-entropy loss during the above-mentioned cross-validation. The five models are ensembled by averaging their predictions. We name the resulting model “*ClipAssistNet*.”

ClipAssistNet outperforms any single Resnet classifiers it is composed of (Table 3). These performances are measured on the test set, that is, on data which was not previously seen by the single neural network classifiers.

**Fig. 3:**
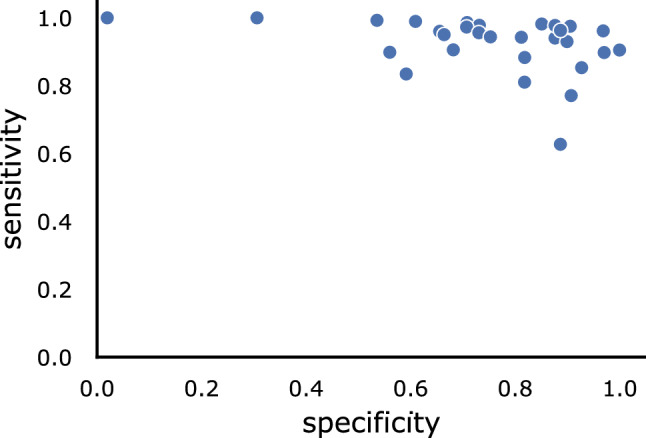
**Besides a few outliers, ClipAssistNet achieves good performance on each video.** Each dot represents the specificity and sensitivity of ClipAssistNet on a given video in the test set. The performance across videos is measured with the same threshold. That is, the median (across videos) of the thresholds providing 95% sensitivity

We further assess the model performance on each video. In particular, we compute the specificity/sensitivity of the model for each test video using the same threshold: the median of all 95% sensitivity thresholds for each video. Besides a few outliers, the model presents good specificity and sensitivity across all test videos (Fig. 3). This is reflected by the higher median specificity across videos (Table 3) compared to the specificity computed across all frames.

**Table 3:** **ClipAssistNet outperforms the single classifiers it is composed of.**These performance metrics are measured on the previously unseen test set

	AUROC	Specificity at 95% sensitivity	Median specificity across videos (at 95% sensitivity)
ClipAssistNet	0.9107	0.6615	0.8120
Single Resnet classifiers (with BCE loss)	0.8929±0.0021	0.6022±0.0131	0.7144±0.0575

To enable the use of our model prediction in an Operating Room setting, we implement ClipAssistNet on an embedded computing board (NVIDIA Jetson AGX Xavier). Despite requiring inferences from 5 submodels, the ensembled model is able to deliver predictions at 16 FPS on this embedded board. This is sufficient for real-time use.

## Discussion

In this work, we present a novel application of computer vision to monitor safety in laparoscopic cholecystectomies.

We propose to increase the safety during clipping time by enforcing the adequate visibility of the clip applier through an intra-operative feedback. This feedback aims at ensuring that the cystic artery/duct are adequately clipped, i.e., correctly encircled by the clipper jaws, while avoiding unintentional interaction with surrounding tissues.

We approach this novel application as an image classification problem, which predicts the tip visibility of the clip applier in each frame. To this end, we prepare a new dataset tailor-made for our application and present a corresponding neural network image classifier.

Our feedback is based on the assumption that enforcing the clipper tip visibility increases the safety of the clipping. To confirm this hypothesis, a board certified surgeon reviewed the safety of 337 clipping actions from 70 of our surgical videos. Only 67.7% of these actions were labeled as safe. The surgeons had visibility of the clipper tip prior to clipping in 37.7% of these safe actions. Nevertheless, 97.6% of all action in which the surgeon proceeded with clipper tip visibility were considered as safe. While obtaining the clipper tip visibility is not the only way to achieve safety, it does bring the surgeon on the safe side and can be considered as best practice. In this respect, our model is also ideal in training situations.

The present work proposes a model to detect the clipper tip visibility during the clipping phase. Several questions still remain on how to integrate the ClipAssistNet predictions in a surgical workflow. For example, the feedback could either be turned on manually or automatically using an algorithm for detecting surgical phases [10]. In any case, the model presents only few spurious detections in the absence of the clipper tip, e.g., in non-clipping phases (Fig. S 1 in Online resources).

More importantly, the optimal threshold for intraoperative use still needs to be determined. The presented specificity values at 95% sensitivity are taken as an example. Slightly lower thresholds might offer better compromise between safety (high sensitivity) and usability (high specificity). Choosing the threshold might require a separate usability study.

Our model is trained on laparoscopic cholecystectomy videos with frame-wise clipper tip visibility annotations. Compared to pixel-wise annotations (e.g., bounding boxes or segmentation), frame-wise annotations can be performed faster. This enables us to swiftly label a large amount of videos (300).

We tailor the annotations guideline for our use case, keeping in mind safety issues. In particular, we label the clipper tip as invisible in case of poor visibility frames. Despite all our effort, a part of subjectivity remains in the annotations of the tip visibility. Especially in case of poor contrast frames, the tip visibility is left to the appreciation of each annotator. Therefore, there subsists grey zone delimiting the tip visibility in these frames. The low agreement across annotators on triply annotated frames (18% of the test frames had no full agreement) highlights this issue. This high labelling noise could potentially impact (i) the trained model and (ii) the measured performance metrics.

We mitigate the impact of the labelling noise on the performance metrics by triple labelling the frames in the test set. We could possibly further reduce the labelling error on the test set by annotating each frames a fourth or a fifth time.

We train deep convolutional neural network classifiers with different losses on this dataset. The neural networks learn to detect the tip of the clip appliers as illustrated by the class activation maps (Fig. 2). If the tip is absent (for example in the absence of the clipper), the classifiers consider it as non-visible.

As mentioned above, the training data are not multiply annotated and probably entails incorrect labels. Forward label noise correction [25] does not improve the model performance. Nevertheless, deep neural networks have been shown to be robust to labelling noise [28]. In fact, for similar label noise level as we observe (7%), deep learning classifiers performance have been shown to be unaffected when using traditional cross-entropy loss (Fig. 1 in [25]).

We substantially improve the tip visibility classification performance by ensembling several models trained during cross-validation. The resulting ensembled model, ClipAssistNet, achieves an overall specificity of 66.15% for a sensitivity at 95%. When computed per video, median specificity across videos raises to 81.2% for a median sensitivity of 95%. This means that ClipAssistNet would correctly throw a warning in 95% of the tip invisible cases; for most videos, 8 out of 10 of these warnings would be correct.

A video example of ClipAssistNet’s prediction during clipping actions can be seen in Video SI 3 in Online resources.

Importantly, ClipAssistNet remains light-weighted enough to run in real-time on an embedded computing board. This is a requirement for delivering the feedback intra-operatively.

## Conclusions

In the present work, we propose a novel intra-operative safety feedback during laparoscopic cholecystectomy. Specifically, this feedback warns surgeons on poor visibility of the clipper tip while clipping of the cystic duct or artery. Our approach is accurate and can run in real-time, a requirement for intra-operative use. We believe this feedback can increase surgeons’ attentiveness when departing from safe visibility of their instrument during this critical phase of laparoscopic cholecystectomy.

## Supplementary Information

Below is the link to the electronic supplementary material.

Supplementary material 1 (mp4 51293 KB)

Supplementary material 2 (mp4 29904 KB)

Supplementary material 3 (mp4 35821 KB)

Supplementary material 4 (pdf 154 KB)

## Data Availability

The data used in this study are under a non-published license and are not publicly available.
