# Supplementary material for: ClipAssistNet: bringing real-time safety feedback to operating rooms
Source: Int J Comput Assist Radiol Surg. 2021 Jul 23;17(1):5–13. doi: 10.1007/s11548-021-02441-x (PMC8739308; doi:10.1007/s11548-021-02441-x)
Supplement: Supplementary file 4 — Supplementary material 4 (pdf 154 KB) [file 11548_2021_2441_MOESM4_ESM.pdf]

ClipAssistNet: bringing real-time safety feedback to operating rooms
Supplementary material

Florian Aspart · Jon L. Bolmgren · Joël L. Lavanchy · Guido Beldi · Michael S. Woods · Nicolas Padoy · Enes Hosgor

F. Aspart, J.L. Bolmgren, M.S. Woods, E. Hosgor
Caresyntax GmbH, Komturstraße 18A, 12099 Berlin, Germany
E-mail: florian.aspart@caresyntax.com

J.L. Lavanchy, G Beldi
Department of Visceral Surgery and Medicine, Inselspital, Bern University Hospital, University of Bern, 3010 Bern, Switzerland

N. Padoy
ICube, University of Strasbourg, CNRS, IHU Strasbourg, France

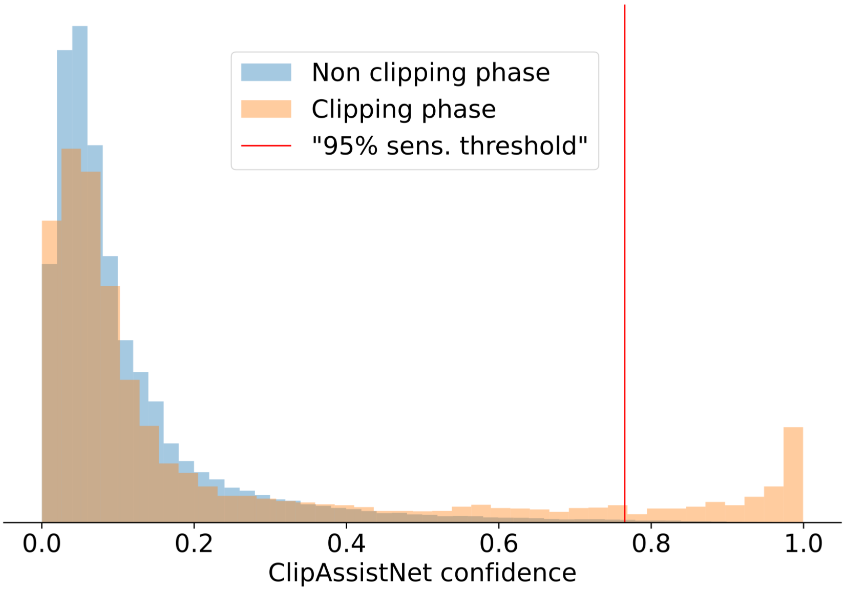

Fig. S 1 ClipAssistNet does not predict the clipper tip being visible in the absence of clipper.
The graph displays the distribution of the model confidence of a frame containing a visible clipper tip (a confidence of 1 corresponds to the clipper tip being visible). The confidence distributions are computed over all videos at 1FPS. The blue distribution corresponds to non-clipping phases, that is to surgical phases not containing a clipper.

Video SI 1 Examples of unsafe clipping. The safety of the clipping is rated post-operatively by a board certified surgeon.

Video SI 2 Examples of clipping with visibility of the clipper tip.

Video SI 3 Video example of ClipAssistNet predictions during clipping actions in the test videos. The green (respectively red) dot in the top left corner corresponds to a tip visible (resp. invisible prediction)
